# Influences of Satisfaction with Telecare and Family Trust in Older Taiwanese People

**DOI:** 10.3390/ijerph110201359

**Published:** 2014-01-27

**Authors:** Chung-Hung Tsai, Yu-Ming Kuo, Shu-Lin Uei

**Affiliations:** 1 Department of Health Administration, Tzu Chi College of Technology, 880, Sec. 2, Chien-Kuo Road, Hualien, Taiwan 97005, China; E-Mail: tzustu@gmail.com; 2 College of Nursing, Kaohsiung Medical University, 100, Shih-Chuan 1st Road, Kaohsiung, Taiwan 80708, China; E-Mail: mchwsl@gmail.com

**Keywords:** older people, telecare, satisfaction, trust, continued use intention

## Abstract

The level of trust given towards telecare by the family members of older people using the service is extremely important. Family trust may be an influential factor in deciding whether to use such services. This study focuses on older people’s satisfaction with telecare and examines their family’s trust in telecare services. Influences on intention to continue using telecare services are also explored. A questionnaire-based survey on 60 communities dwelling older people who had been receiving telecare services in the past two years was employed. This study developed a satisfaction and trust scale based on previous studies. Our results show that older people’s satisfaction with telecare services and families’ trust were influential in decided whether to continue to use of telecare services. These findings can help medical institutions to better insight into the user experience of telecare to help them provide future services that better comply with clients’ desires and requirements.

## 1. Introduction

Recent improvements in medical care and living standards have increased the average life expectancy of Taiwanese people, leading to an ageing population. This potentially creates numerous medical problems and may influence lifestyle transitions.

These factors have led to increased attention to medical care issues, care of disabled people, emergency medical services, chronic diseases and medication complications. Telecare in health is an emerging field of remote medical technology, which has seen rapid development in recent years. Through information and communication technology, telecare monitors physical health and activities, provides safety and security and enables more convenient information and interpersonal communication [1,2,3,4]. It also provides and conveys health and social care directly into clients’ homes [5]. Telecare allows older people to be cared for in their own family home environments, this has been shown to increase older people’s autonomy and levels of self-esteem [6]. To date, most telecare studies have focused on the scientific dimensions of the service. However, a few studies have investigated the influence of information technology services in the care of older people, focusing on health related quality of life of clients [1,2,3,4,7,8,9].

This study is based on the Theory of Reasoned Action (TRA). TRA provides a model predicting the intention to perform a behaviour based on an individual’s attitudinal and normative beliefs [10]. A change in any of these factors results in a different behavior being explained. DeLone and McLean [11] have shown that satisfaction with a service has a positive impact in relation to continued use. Mayer and Davis [12] viewed this form of belief of trust to directly affect an individual’s intention to use a service. It is expected these relationships will apply in the delivery of telecare services. In this work, this theory promotes that the belief of trust (perceptions of telecare attributes) could cause attitudes (satisfaction), and influenced intention to reuse (continued use intention) [10].

All participants in this study lived in Hualien County, in Eastern Taiwan. The majority (80%) of this geographical region is covered by mountains [13]. Medical resources are not evenly distributed in Hualien Couny, the majority of medical resources are delivered in urban areas. These geographic traits, make it problematic to deliver conventional medical resources to all the people in this region of Taiwan. Therefore, telecare services may be one potential option to maximize healthcare delivery. The Mennonite Christian Hospital started a telecare service in Eastern Taiwan in 2010. Services include tele-consultation, tele-physiological monitoring, tele-health education, medication safety, alarm systems, and life resource linkage. In this study, services are categorized into the fields of telecare, telehealth and telemedicine. Tele-physiological monitoring is the most commonly used within our hospital and this will form the focus of this study.

There are four distinct aims within this study: (a) determine the telecare service(s) most commonly used by older people; (b) older people’s satisfaction with telecare; (c) the trust of older people’s families towards telecare services; and (d) influences of older people’s satisfaction with telecare services and their families’ trust on the intention to continue using the service. From these four aims, specific attention will be given to family trust towards telecare. This is extremely important as older people are the ones who are cared for, but the family (who are the care givers) making the critical decision as to whether to continue or stop the use of telecare services.

## 2. Methods

This study conducted surveys and adopted convenience sampling. The Institutional Review Board (IRB) of Mennonite Christian Hospital sanctioned this study.

### 2.1. Participants

In this study, a convenience sample was taken from the sole provider of telecare in a hospital in Eastern Taiwan. The foundation of the hospital aims to provide home care services for patients in this remote mountainous region. The inclusion criteria for the sample in this study are as follows: (a) users of telecare or telehealth services during the previous two years; (b) have paid for the telecare or telehealth services (c) family members do not live with the telecare or telehealth user.

### 2.2. Measures

A specifically designed telecare satisfaction scale was developed. This was based on themes or dimensions developed in previous telecare studies studies [14,15].

Our scale comprised five items for the older person using telecare in health care. In addition, we adopted the telecare trust scale for families developed by Shea and Effken [16] and Varghese and Phillips [17]. The scale of continued use intention was based on the studies that used telecare systems [2,18]. For each of these three dimensions see the questionnaire provided in the Appendix.

The questionnaire was made up three dimensions and includes twelve five-point Likert scale questions. For each question a score of one equates to strongly disagree and a score of five means strongly agree. Three investigators collected data through telephone interview or by visiting the user’s, this involved oral questioning by investigators and completion of the questionnaire. Professional training was given to all three investigators and this included training with the interview protocol to ensure rigor in the study and to confirm that all data was correctly collected. Data collection took six months to complete.

### 2.3. Method

The relationship between older people’s satisfaction with telecare and their intention to continue to use this service was verified. Firstly, we calculated the satisfaction and their intention to continue using the service. Average scores of the questionnaires were calculated, and then SPSS 18 was used to perform linear regression analysis of the sample. Linear regression analysis was conducted to determine the effect of family trust on intention to use telecare services in the future.

## 3. Statistical Analysis

### 3.1. Descriptive Statistical of the Respondents

From 243 people using telecare services in the region there were 60 older people who met the study inclusion criteria. A total of sixty fully completed questionnaires were collected, giving a response rate of 100%. Additionally, the family members of sixty telecare service users also completed level of trust questionnaires; again the response rate was 100%. The demographic characteristics of telecare users showed that the average user age was 65 ± 4.5. Of the participants, 67% were female; 80% had received nine years or less of formal education; half the sample were married and 40% were widowed; 71.7% lived with their family and 28.3% lived alone; 45% spent most of their daytime hours alone at home and 53.3% lived in the mountains area. In relation to health status, 85% of the sample suffered from two or more chronic diseases.

### 3.2. Use of Telecare Services by Older People

The first question of this study was used to identify the most popular telecare services amongst older people. The results show that the most commonly used telecare services by the sixty participants were, in descending order: tele-physiological monitoring (100%), tele-healthcare education (76.7%), tele-healthcare management (63.3%), offering living resources (50%), tele-healthcare consultation (15%), tele-medication safety advisement (13.3%), and remote video visitation (5%).

### 3.3. Satisfaction of Older People Using Telecare Services

Table 1 shows the overall satisfaction scores of older people accessing telecare services. Telecare users scored an average of 4.25 regarding overall service quality. The highest-rated item was “you think your health condition improved after using telecare services”, with an average satisfaction of 3.91. The lowest-rated item was “telecare reduces your expenditures on health care”, with an average satisfaction score of 3.51 reported.

**Table 1:** Satisfaction scores.

Item	Minimum	Maximum	Mean	Variance
You think your health condition improved after using telecare services	3	5	3.91	0.547
Telecare reduces your care time for health issues	3	5	3.66	0.573
Telecare alleviates your concerns with health	1	5	3.81	0.706
Telecare reduces your expenditures on health care	2	5	3.51	0.737
Telecare has superior overall service quality	3	5	4.25	0.365

### 3.4. Family Trust towards Telecare Services

In addition to the examination of telecare from the users’ perspective, this study also examined levels of family trust towards telecare services. Table 2 demonstrates that families had an average trust score of 3.31 with the quality of the telecare service, which was the highest score reported across all dimensions as was older people’s satisfaction with telecare services. The item with the lowest average trust was “You believe that telecare saves care time for your family”, with an average of 2.63.

### 3.5. Older Peoples’ Satisfaction and Intention to Continue to Use Telecare Services

Statistical results reveal that older peoples’ satisfaction with their telecare services has a positive impact on their desire to continued using the service (Table 3). Hence, a higher telecare service satisfaction led to a higher intention to continue using telecare services. The *t*-value was 6.745 and adjusted R^2^ was 0.434, which implied good explanatory power for the overall model. There appears no significant relationship exists between age and satisfaction.

**Table 2:** Descriptive statistics of families’ trust in telecare.

Item	Minimum	Maximum	Mean	Variance
You believe that telecare enhances your family’s health conditions	1	4	2.86	0.42
You believe that telecare saves care time for your family	1	4	2.63	0.42
You believe that telecare alleviates your concerns with the health conditions of your family	1	5	3.06	0.41
You trust the quality of telecare services	1	5	3.31	0.4

**Table 3:** Older peoples’ satisfaction and the continued use intention.

Control Variables	Continued Use Intention
Age	0.068
Satisfaction (Elderly)	0.666 ^**^
Max VIF	1.232
*t*-value	6.745
Adjusted R^2^	0.434

Note: ^**^ *p* < 0.01.

### 3.6. Family Trust in Telecare and Continued Use Intention

In this study, statistical results demonstrated that family trust had a positive impact on the intention to continue using telecare services (Table 4). 

**Table 4:** Family trust and the intention to continue using telecare.

Control Variables	Continued Use Intention
Age	0.086
Trust (Family)	0.430 ^**^
Max VIF	1.009
*t*-value	3.804
Adjusted R^2^	0.317

Note: ^**^ *p* < 0.01.

It would appear that a higher level of family trust in telecare services leads greater intention for older people to continue to use the service. The *t*-value was 3.804 adjusted R^2^ was 0.317, which implied good explanatory power for the overall model. There was no significant relationship between age and satisfaction. Thus, this model may be appropriate to explain factors that influenced the intention to continue paying for telecare services.

## 4. Discussion

Globally, information and communication technology products that are used in telecare services have developed greatly in recent years. This is no exception in Taiwan where implementing telecare technology has become increasingly popular. Nonetheless, considering the future development of telecare services from a purely scientific and technical perspective creates a potential gap between ideals and users’ regular life [1,2,3,4]. 

In Taiwan, many older people cannot read and have no previous experience of using high-tech products. Therefore, this study focused on the user experiences and families’ trust in telecare. These results provide a referential framework for manufacturers of medical instrument and to the medical institutions to help improve design of relevant healthcare services for older people.

Our results reveal that tele-physiological monitoring, tele-healthcare education, and tele-healthcare management were the most commonly used items by our study participants. In addition, tele-healthcare consultation, tele-medication safety advice, and remote video visitations were the most rarely used items. Infrequent use of remote video visitations may be potentially explained by the fact that Taiwanese families are inclined to dwell together or in adjacent neighborhoods. With this cultural background, the elderly can quickly and easily get help from others. In addition, we believe that telecare services are still in a developmental phase in Taiwan, and medical institutions could easily promote and produce less complex medical services. We also found that users were not used to using tele-healthcare consultations and tele-medication safety advice and were inclined to go to the hospital in person. We suggest that reference is made to the work of Turner *et al*. [19] on the development of future telecare studies. This would ensure the perceived perspective of the user influences further exploration of use of healthcare services.

In this study, the mean score of older people’s overall satisfaction levels with telecare was 4.25, indicating that telecare services mainly satisfied their needs. Items concerning satisfaction showed that older people had lower expenditures on telecare services than on other items. This implies that cost may be a significant factor that influences users’ decision to continue using the service. Whitten *et al*. [20] contended that in addition to superior functional capability, finance is the essential key factor for a telemedicine service to succeed. Therefore, future research should focus on how telecare services can balance the revenue of medical institutions and user costs.

Family trust mean scores for telecare was 3.31, which was much lower than overall telecare satisfaction scores given by older people. This may have resulted as families were not directly using the service and may not have been present when their relative used the service. Therefore, they could not fully realize the quality and potential benefits of telecare. In addition, telecare services do not allow physicians to make a medical diagnosis or to prescribe for clients under medical regulation in Taiwan. Thus, time spent on taking care of the older people can only be partially reduced, however the client still has to use hospital service and is required to be taken to the hospital by the family for the purpose of diagnosis and prescription. Another probable cause is Taiwan’s emphasis on the concept of filial piety, which indicates that children have the responsibility and obligation to care for their frail elderly and should not rely solely on technology. We suggest that future research should examine varying medical systems in different societies and cultures to gain insight as to whether these factors result in varying usage habits and perceptions of telecare.

Finally, by using regression analysis, this study verified the influence of older people’s satisfaction with telecare services and families’ trust on their intention to continue using the service. According to our results, these two factors had a positive and significant correlation with the intention to continue use. This corresponds to previous studies examining satisfaction with telecare services [1,2,3,4,21,22]. Furthermore, these results can be used to discuss the influence of family trust on the use intention of telecare and can broaden the explanatory scope of preceding studies by showing that families’ trust in telecare services is a critical factor that supports users to continue using the services. Historically, TRA theory has been applied to to the examination patients or their families perspective [23]. Most of studies have used the TAM model [24]. We suggest that in addition to providing services for users, consideration of feelings of their significant others should be made by medical institutions, because older people (whose physiological functional capability may have deteriorated) may not be in best position to fully express their needs. This highlights the importance of the role the family plays in this decision making process and feedback may potentially have an influence on the dependence of the older person.

However, in remote mountainous areas or remote islands in Taiwan, insufficient medical resources contribute to the thriving development of telecare. This study provides valuable information for medical institutions who use telecare services. Understanding the users’ perspective and experience will potentially allow them to tailor services to best meet these needs.

### Limitations

Related samples are difficult to access because only a few Taiwan medical institutions promote home telecare systems, and some of these institutions may still be operating in an experimental phase and are not appropriate for comprehensive testing. This study experienced limitations in the conceptualization and expansion of the theoretical model because our samples were from rural area in Eastern Taiwan, where cases could not be monitored continuously, samples were insufficient, and the vastness of the area impeded the distribution of questionnaires. The valid samples from this study were all elderly people who had used telecare services for at least two years. The conclusion is that a high degree of satisfaction in the service influenced continued use of telecare. However, participants in the study were recruited from an area where there is uneven distribution. Another potential limitation is the restricted time and space the patients had to use telecare. However, this study does still provide a valuable and unique insight into the existing knowledge on use of telecare services in older people.

We recommend future research target people who have chosen to discontinue their use of telecare services to evaluate their satisfaction and ascertain why they have chosen to discontinue use. In addition, recommendation is made that any future research be able to continuously track telecare users, as presently there remains a gap between intention to continue using telecare services and continued use behavior in this area. This phenomenon can be explored from the normalization process theory which may be a new technology becomes routinely embedded in a social context as the result of individual [25].

## 5. Conclusions

Most telecare studies to date have focused on European and American populations. This study provides a different perspective as it is one of only a few studies which has examined telecare use in older Chinese people. As most Asian countries are densely populated, even with technological developments there is no guarantee that Asian people are familiar with and using telecare services. Our findings are as follows: (1) tele-physiological monitoring, tele-healthcare education, and tele- healthcare management were the most commonly used items; (2) Taiwanese older people were satisfied with telecare services; (3) we verified the influence of older people’s satisfaction with telecare services and families’ trust on the continued use intention. This study provides greater understanding and expands the explanatory scope of the existing literature.

## Appendix

Satisfaction scale (Cronbach α = 0.912; [14,15])

1. You believe your health condition has improved following the use of telecare services.
2. Telecare reduces the time you spend on health-realated issues.
3. Telecare alleviates health-realated concerns.
4. Telecare reduces the cost of health care.
5. Telecare has superior overall service quality.

Trust scale (Cronbach α = 0.899; [16])

1. You believe that telecare enhances your family’s health conditions.
2. You believe that telecare saves care time for your family.
3. You believe that telecare alleviates your concerns with your family’s health conditions.
4. You trust the quality of telecare services.

Continued use intention (Cronbach α = 0.931; [2,18])

1. When I am in need, I will use a telecare system.
2. I will continue using the telecare system.
3. I have high intention to use the telecare system.
